# Tuberculosis — United States, 2016

**DOI:** 10.15585/mmwr.mm6611a2

**Published:** 2017-03-24

**Authors:** Kristine M. Schmit, Zimy Wansaula, Robert Pratt, Sandy F. Price, Adam J. Langer

**Affiliations:** ^1^Epidemic Intelligence Service, CDC; ^2^Division of Tuberculosis Elimination, National Center for HIV/AIDS, Viral Hepatitis, STD, and TB Prevention, CDC.

In 2016, a total of 9,287 new tuberculosis (TB) cases were reported in the United States; this provisional* count represents the lowest number of U.S. TB cases on record and a 2.7% decrease from 2015 (*1*). The 2016 TB incidence of 2.9 cases per 100,000 persons represents a slight decrease compared with 2015 (-3.4%) (Figure). However, epidemiologic modeling demonstrates that if similar slow rates of decline continue, the goal of U.S. TB elimination will not be reached during this century (*2*). Although current programs to identify and treat active TB disease must be maintained and strengthened, increased measures to identify and treat latent TB infection (LTBI) among populations at high risk are also needed to accelerate progress toward TB elimination.

**FIGURE:**
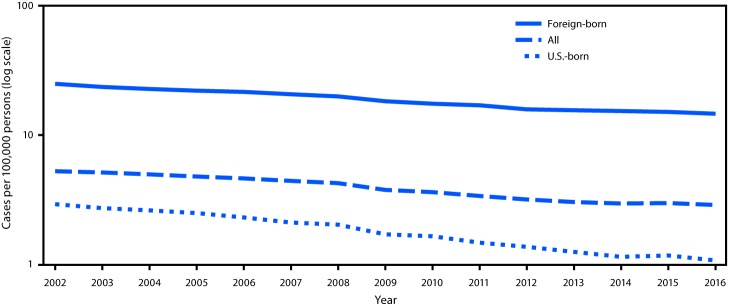
Tuberculosis (TB) incidence overall and among U.S.-born and foreign-born persons* — United States, 2002–2016 * U.S.-born persons are those persons who were born in the United States or a U.S. island area or were born abroad to a U.S. citizen parent or parents. All others, including naturalized U.S. citizens, are considered foreign-born persons.

Health departments in the 50 states and District of Columbia (DC) electronically report to CDC^†^ verified cases of TB that meet the CDC and Council of State and Territorial Epidemiologists case definition for TB. Reports include demographic and clinical information, as well as medical and social risk factors for TB disease. Persons reported with TB are classified as U.S.-born or foreign-born persons based on established criteria^§^; race/ethnicity is self-reported. U.S. Census Bureau midyear population estimates provide the denominators used to calculate TB incidence overall (*3*). The Current Population Survey (https://www.census.gov/programs-surveys/cps.html) provides the population denominators used to calculate TB incidence according to national origin and racial/ethnic group.

State-specific TB incidence in 2016 ranged from 0.2 cases per 100,000 persons in Wyoming to 8.3 in Hawaii (median state incidence = 1.9). Twelve states (Alaska, Arkansas, California, Florida, Georgia, Hawaii, Maryland, Minnesota, New Jersey, New York, North Dakota, and Texas) and DC reported incidence higher than the national incidence (Table 1). As in previous years, four states (California, Florida, New York, and Texas) reported >500 cases each in 2016, accounting for 50.9% of reported cases nationwide.

**TABLE 1:** Tuberculosis (TB) case counts and incidence with annual percent changes, by U.S. Census division and state/district — United States and the District of Columbia, 2015 and 2016

Census division/state	Case count*			Incidence		
	2015	2016	% Change	2015	2016	% Change^†^
**Division 1: New England**						
Connecticut	70	52	-25.7	2.0	1.5	-25.5
Maine	18	23	27.8	1.4	1.7	27.6
Massachusetts	192	190	-1.0	2.8	2.8	-1.4
New Hampshire	13	15	15.4	1.0	1.1	15.0
Rhode Island	30	12	-60.0	2.8	1.1	-60.0
Vermont	7	5	-28.6	1.1	0.8	-28.4
**Total**	**330**	**297**	**-10.0**	**2.2**	**2.0**	**-10.2**
**Division 2: Middle Atlantic**						
New Jersey	326	294	-9.8	3.6	3.3	-9.9
New York	763	768	0.7	3.9	3.9	0.7
Pennsylvania	200	174	-13.0	1.6	1.4	-12.9
**Total**	**1289**	**1236**	**-4.1**	**3.1**	**3.0**	**-4.1**
**Division 3: East North Central**						
Illinois	343	342	-0.3	2.7	2.7	0.0
Indiana	116	109	-6.0	1.8	1.6	-6.3
Michigan	131	133	1.5	1.3	1.3	1.4
Ohio	143	141	-1.4	1.2	1.2	-1.5
Wisconsin	69	40	-42.0	1.2	0.7	-42.1
**Total**	**802**	**765**	**-4.6**	**1.7**	**1.6**	**-4.6**
**Division 4: West North Central**						
Iowa	38	48	26.3	1.2	1.5	25.8
Kansas	36	39	8.3	1.2	1.3	8.3
Minnesota	150	168	12.0	2.7	3.0	11.2
Missouri	92	101	9.8	1.5	1.7	9.5
Nebraska	33	28	-15.2	1.7	1.5	-15.7
North Dakota	9	22	144.4	1.2	2.9	144.1
South Dakota	17	12	-29.4	2.0	1.4	-30.0
**Total**	**375**	**418**	**11.5**	**1.8**	**2.0**	**11.0**
**Division 5: South Atlantic**						
Delaware	22	16	-27.3	2.3	1.7	-27.9
District of Columbia	33	25	-24.2	4.9	3.7	-25.4
Florida	602	639	6.1	3.0	3.1	4.3
Georgia	320	302	-5.6	3.1	2.9	-6.6
Maryland	176	220	25.0	2.9	3.7	24.6
North Carolina	199	220	10.6	2.0	2.2	9.3
South Carolina	104	103	-1.0	2.1	2.1	-2.3
Virginia	212	205	-3.3	2.5	2.4	-3.8
West Virginia	10	14	40.0	0.5	0.8	40.8
**Total**	**1678**	**1744**	**3.9**	**2.7**	**2.7**	**2.7**
**Division 6: East South Central**						
Alabama	119	112	-5.9	2.5	2.3	-6.1
Kentucky	67	91	35.8	1.5	2.1	35.4
Mississippi	74	61	-17.6	2.5	2.0	-17.5
Tennessee	131	103	-21.4	2.0	1.5	-22.0
**Total**	**391**	**367**	**-6.1**	**2.1**	**1.9**	**-6.5**
**Division 7: West South Central**						
Arkansas	90	91	1.1	3.0	3.0	0.8
Louisiana	119	127	6.7	2.5	2.7	6.4
Oklahoma	67	78	16.4	1.7	2.0	15.9
Texas	1333	1250	-6.2	4.9	4.5	-7.7
**Total**	**1609**	**1546**	**-3.9**	**4.1**	**3.9**	**-5.1**
**Division 8: Mountain**						
Arizona	198	188	-5.1	2.9	2.7	-6.6
Colorado	73	64	-12.3	1.3	1.2	-13.8
Idaho	11	18	63.6	0.7	1.1	60.7
Montana	9	4	-55.6	0.9	0.4	-56.0
Nevada	85	56	-34.1	2.9	1.9	-35.4
New Mexico	47	39	-17.0	2.3	1.9	-17.0
Utah	37	20	-45.9	1.2	0.7	-47.0
Wyoming	4	1	-75.0	0.7	0.2	-75.0
**Total**	**464**	**390**	**-15.9**	**2.0**	**1.6**	**-17.2**
**Division 9: Pacific**						
Alaska	68	57	-16.2	9.2	7.7	-16.6
California	2130	2073	-2.7	5.5	5.3	-3.3
Hawaii	127	119	-6.3	8.9	8.3	-6.5
Oregon	76	70	-7.9	1.9	1.7	-9.4
Washington	207	205	-1.0	2.9	2.8	-2.7
**Total**	**2608**	**2524**	**-3.2**	**5.0**	**4.8**	**-4.1**
**United States**	**9546**	**9287**	**-2.7**	**3.0**	**2.9**	**-3.4**

* Case counts based on data from the National Tuberculosis Surveillance System as of February 17, 2017. U.S. Census Bureau midyear population estimates provide the denominators used to calculate TB incidence.

^†^ Percentage change in incidence is calculated on the basis of unrounded incidence for 2015 and 2016.

Among 9,287 TB cases reported in 2016, U.S.-born persons accounted for 2,935 (31.6%) cases, and 6,307 (67.9%) cases occurred among foreign-born persons; 45 (0.5%) cases occurred among persons whose national origin was not known (Table 2). TB incidence among U.S.-born persons (1.1 cases per 100,000) decreased 8.4% from 2015 (Figure). Incidence among foreign-born persons (14.6 cases per 100,000) decreased 3.2% from 2015, but was approximately 14 times the incidence among U.S.-born persons.

**TABLE 2:** Tuberculosis (TB) case counts and incidence,* by national origin and race/ ethnicity — United States, 2013–2016†

U.S. population group	Case count (incidence)			
	2013	2014	2015	2016
**U.S.-born^§^**				
Hispanic	650 (1.8)	651 (1.8)	657 (1.8)	599 (1.6)
White, non-Hispanic	1,092 (0.6)	970 (0.5)	987 (0.5)	911 (0.5)
Black, non-Hispanic	1,251 (3.6)	1,186 (3.4)	1,139 (3.3)	1,062 (3.0)
Asian	151 (2.4)	137 (2.1)	136 (2.1)	145 (2.1)
American Indian/Alaska Native	122 (5.6)	114 (5.1)	144 (7.0)	108 (5.0)
Native Hawaiian/Pacific Islander	45 (6.3)	83 (12.4)	88 (12.7)	67 (9.2)
Multiple or unknown race/ ethnicity	44 (—^¶^)	37 (—^¶^)	35 (—^¶^)	43 (—^¶^)
**Total U.S.-born**	**3,355 (1.2)**	**3,178 (1.2)**	**3,186 (1.2)**	**2,935 (1.1)**
**Foreign-born**				
Hispanic	2,033 (11.1)	2,093 (11.2)	2,033 (10.4)	1,979 (10.0)
White, non-Hispanic	323 (4.2)	279 (3.6)	252 (3.3)	293 (3.9)
Black, non-Hispanic	835 (24.5)	828 (23.6)	852 (23.0)	898 (22.3)
Asian	2,850 (29.0)	2,920 (29.3)	3,089 (29.0)	3,023 (26.9)
American Indian/Alaska Native	2 (3.0)	—**	1 (1.9)	2 (5.8)
Native Hawaiian/Pacific Islander	17 (6.7)	8 (3.6)	14 (4.3)	12 (3.3)
Multiple or unknown race/ ethnicity	125 (—^¶^)	92 (—^¶^)	111 (—^¶^)	100 (—^¶^)
**Total foreign-born**	**6,185 (15.6)**	**6,220 (15.4)**	**6,352 (15.1)**	**6,307 (14.6)**
**Unknown national origin**	9 (—^¶^)	5 (—^¶^)	8 (—^¶^)	45 (—^¶^)
**Total**	**9,549 (3.0)**	**9,403 (3.0)**	**9,546 (3.0)**	**9,287 (2.9)**

* Incidence calculated per 100,000 persons.

^†^ Case counts based on data from the National Tuberculosis Surveillance System as of February 17, 2017. The Current Population Survey (https://www.census.gov/programs-surveys/cps.html) provides the population denominators used to calculate TB incidence according to national origin and racial/ ethnic group.

^§^ U.S.-born persons were born in the United States or a U.S. island area or born abroad to a U.S. citizen parent or parents. All others, including naturalized U.S. citizens, are considered to be foreign-born persons.

^¶^ Incidence was not calculated for these categories.

** No cases for foreign-born American Indian/Alaska Native population occurred in 2014.

Among U.S.-born persons, TB incidence remained stable among non-Hispanic whites (0.5 cases per 100,000) and Asians (2.1), but decreased from 2015 in all other racial/ethnic groups including Hispanics (1.6 [-11.4%]), non-Hispanic blacks (3.0 [-6.8%]), American Indian/Alaska Natives (5.0 [-28.8%]), and Native Hawaiian/Pacific Islanders (9.2 [-27.3%]) (Table 2). TB incidence has decreased or remained stable since 2013 in all U.S.-born racial/ethnic groups except American Indian/Alaska Natives and Native Hawaiian/Pacific Islanders, which experienced increases during this period before decreasing in 2016.

Among foreign-born persons, the highest TB incidence in 2016 was among Asians (26.9 cases per 100,000), followed by non-Hispanic blacks (22.3) and Hispanics (10.0), and most foreign-born racial/ethnic groups have experienced gradual decreases between 2013 and 2016. The top five countries of origin for foreign-born persons reported with TB disease in the United States were Mexico (1,194 cases, 18.9% of all foreign-born cases), the Philippines (795, 12.6%), India (593, 9.4%), Vietnam (496, 7.9%), and China (383, 6.1%). Cases in persons born in these countries accounted for 54.9% of all cases among foreign-born persons.

HIV status was known for 86.7% of TB cases reported in 2016; among these patients, 5.8% had documented HIV co-infection. Living in congregate settings such as shelters, long-term care facilities, and correctional facilities is a known risk factor for TB exposure (*4*), and complete data on these risk factors were available for >93% of cases. Among these, 4.6% of patients reported having experienced homelessness in the year preceding diagnosis. In addition, 1.8% were reported as residing in a long-term care facility, and 3.5% were reported as being confined in a correctional facility at the time of diagnosis.

The most recent year for which complete drug-susceptibility data are available is 2015; the data include test results for 98.7% of culture-confirmed TB cases. In 2015, 88 cases of multidrug-resistant TB^¶^ occurred; multidrug-resistant TB accounted for 0.4% and 1.2% of culture-confirmed TB cases among U.S.-born and foreign-born persons, respectively. Among the 88 multidrug-resistant TB cases, 72 (81.8%) occurred in persons with no reported prior history of TB disease. In 2015, one case of extensively drug-resistant TB** was reported.

## Discussion

Provisional data for 2016 demonstrate a slight decline in both TB case count and incidence in the United States compared with 2015. However, previously published epidemiologic modeling suggests that maintaining similar rates of decline in the future will not be sufficient to achieve TB elimination in the United States during this century (*2*). Current TB control priorities, including early identification of TB cases, prompt institution of appropriate treatment, and identification of exposed contacts remain critical to preventing a resurgence of TB; to achieve TB elimination, expanded measures and new strategies are needed. Epidemiologic models demonstrate that identifying and treating persons with LTBI (a condition that occurs when a person is infected with *Mycobacterium tuberculosis* without signs and symptoms, or radiographic or bacteriologic evidence of TB disease) is critical to accomplishing the goal of TB elimination (*2*). This strategy is consistent with CDC recommendations as well as 2016 recommendations from the U.S. Preventive Services Task Force (USPSTF) to screen for LTBI with tests such as the tuberculin skin test or interferon-gamma release assay in populations that are at increased risk for TB (*4*,*5*). The USPSTF characterizes populations at increased risk as those persons who were born in, or formerly resided in, countries with increased TB prevalence as defined by the World Health Organization (WHO) (*6*); and persons who currently live in, or have lived in high-risk congregate settings such as homeless shelters, correctional facilities, and long-term care facilities.

In 2016, four of the top five countries of origin for foreign-born persons reported with TB disease were considered high TB burden countries by WHO (China, India, Philippines, Vietnam), and accounted for 36% of incident TB cases among foreign-born persons in the United States (*6*). Because approximately 90% of TB cases in foreign-born persons in the United States are attributable to reactivation of LTBI, targeted testing for and treatment of LTBI among foreign-born persons from countries with high TB prevalence could be an effective strategy to decrease TB incidence (*7*). The current recommendation from the USPSTF to test persons at increased risk regardless of length of time in the United States is in keeping with evidence that reactivation of LTBI remains a substantial concern, even in foreign-born persons who have lived in the United States for many years (*8*,*9*).

Workers in high-risk congregate settings are also at increased risk for TB and should be included as part of a targeted testing and treatment approach (*4*). Other persons at risk for TB infection or for progression from LTBI to TB disease who should also be included in this strategy include close contacts of infectious TB patients, persons with immunosuppression, persons with other medical conditions (e.g., diabetes mellitus, chronic renal failure, or silicosis) associated with progression from LTBI to TB disease, and persons with fibrotic changes on a chest radiograph suggestive of inactive TB disease (*4*).

The findings in this report are subject to at least two limitations. First, this analysis is limited to reported provisional case counts and incidence rates for 2016. Second, incidence rates are calculated based on estimated population denominators for 2016.

Although TB case counts and incidence are decreasing in the United States, progress is insufficient to achieve in this century the goal of TB elimination. Measures to diagnose and treat active TB disease must continue, and new strategies aimed at accelerating progress toward TB elimination in the United States, such as targeted testing for and treatment of LTBI, should also be employed. Expanded partnerships with health care providers outside of the public health sector will be important in effectively implementing such a strategy.

**Summary**

**What is already known about this topic?**

An annual decline in the number of cases and incidence of tuberculosis (TB) in the United States was found beginning in 1993 and continuing until 2015, when the case count increased and the incidence remained the same as the previous year.

**What is added by this report?**

Provisional data for 2016 indicate a decreased TB case count and incidence compared with 2015.

**What are the implications for public health practice?**

Current strategies are effective in controlling TB, but not sufficient to promote progress toward the goal of eliminating TB in the United States. Current TB control priorities remain important to prevent a resurgence of TB, but expanded measures and new strategies are needed to achieve TB elimination. Targeted testing and treatment of latent TB infection in populations at high risk for TB are key strategies for lowering incidence and moving toward elimination.

## Footnotes

* This report is limited to National Tuberculosis Surveillance System data as of February 17, 2017. Updated data will be available in CDC’s annual TB surveillance report later this year.

† https://www.cdc.gov/tb/programs/rvct/instructionmanual.pdf.

§ U.S.-born persons are defined as persons who were born in the United States or a U.S. island area or born abroad to a U.S. citizen parent or parents. All others, including those who have become U.S. citizens through naturalization, are considered to be foreign-born persons.

¶ Multidrug-resistant tuberculosis (TB) is defined by the World Health Organization as a case of TB in a person with a *Mycobacterium tuberculosis* isolate resistant to at least isoniazid and rifampin.

** Extensively drug-resistant TB is defined by the World Health Organization as a case of TB in a person with a *Mycobacterium tuberculosis* isolate with resistance to at least isoniazid and rifampin among first-line anti-TB drugs, resistance to any fluoroquinolone (e.g., ciprofloxacin or ofloxacin), and resistance to at least one of three second-line injectable drugs (i.e., amikacin, capreomycin, or kanamycin).
